# Do altering in ornithine decarboxylase activity and gene expression contribute to antiproliferative properties of COX inhibitors?

**DOI:** 10.1038/sj.bjc.6600815

**Published:** 2003-04-01

**Authors:** J Ostrowski, T Wocial, H Skurzak, W Bartnik

**Affiliations:** 1 Department of Gastroenterology, Medical Center for Postgraduate Education; Maria Skłodowska-Curie Memorial Cancer Center and Institute of Oncology, ul. Roentgena 5, 02-781 Warsaw, Poland; 2 Department of Immunology, Maria Skłodowska-Curie Memorial Cancer Center and Institute of Oncology, ul. Roentgena 5, 02-781 Warsaw, Poland

**Keywords:** ODC, c-*myc*, *Egr-1*, c-*fos*, apoptosis, proliferation, COX inhibitors

## Abstract

Two isoforms of cyclooxygenase (COX) participate in growth control; COX-1 is constitutively expressed in most cells, and COX-2 is an inducible enzyme in response to cellular stimuli. An induction of COX-2 found in neoplastic tissues results in increased cell growth, inhibition of apoptosis, activation of angiogenesis, and decreased immune responsiveness. Although both COX-1 and COX-2 inhibitors are suppressors of cell proliferation and appear to be chemopreventive agents for tumorigenesis, the molecular mechanisms mediating antiproliferative effect of COX inhibitors are still not well defined. This study contrasts and compares the effects of aspirin and celecoxib, inhibitors of COX-1 and COX-2, in rat hepatoma HTC-IR cells. The following were assessed: cell proliferation and apoptosis, ornithine decarboxylase (ODC) activity, and pattern expression of three immediate-early genes, c-*myc*, *Egr-1*, and c-*fos.* We have shown that the treatment of hepatocytes *in vitro* with the selective COX-2 inhibitor, celecoxib, was associated with induction of apoptosis and complete inhibition of cellular proliferation. Aspirin exhibited a small antiproliferative effect that was not associated with apoptosis. Treatment with celecoxib produced dose- and time-dependent decrease in ODC activity. In addition, at higher drug concentration the decrease in ODC activity was greater in proliferating than in resting cells. Much lesser inhibitory effect on ODC activity was observed in aspirin-treated cells. The two COX inhibitors did not change c-*myc* expression, significantly decreased the expression of *Egr-1*, and differentially altered expression of c-*fos*; aspirin did not change, but celecoxib dramatically decreased the levels of c-*fos*-mRNA. Our study revealed that celecoxib and aspirin share the ability to inhibit ODC activity and alter the pattern of immediate-early gene expression. It seems that some of the observed effects are likely to be related to COX-independent pathways. The precise mechanisms of action of COX inhibitors should be defined before using these drugs for cancer chemopreventive therapy.

Mammalian cells express two related but unique isoforms of cyclooxygenase (COX), COX-1 and COX-2, the rate-limiting enzymes in prostaglandin (PG) biosynthesis; they participate in both normal and neoplastic growth responses (Bennett *et al*, 1977; Maxwell *et al*, 1990; Kokoglu *et al*, 1998; Denkert *et al*, 2001). While COX-1 is a constitutively expressed enzyme that is generally involved in cell function of most tissues, COX-2 is an inducible enzyme in response to cellular stimuli, including mitogens, tumour promoters, cytokines, and other inflammatory mediators (Eberhart and DuBois, 1995; Smith *et al*, 1996; Beejay and Wolfe, 1999; Whittle *et al*, 2000). Although the molecular mechanisms causing the overexpression of COX-2 in various cancer cells are not understood, it is known that increased level of COX-2 results in enhanced PG production.

Both COX-1 and COX-2 inhibitors are suppressors of cell proliferation and appear to be chemopreventive agents for tumorigenesis (Thun *et al*, 1991; Scheinemachers and Everson, 1994; Kawamori *et al*, 1998; Taketo, 1998a,1998b; Elder *et al*, 2000; Joki *et al*, 2000; Lui *et al*, 2000). It is clear that inhibitors of COX-2, but not COX-1, strongly suppress cell growth by inducing apoptosis (Erickson *et al*, 1999; Ding *et al*, 2000; Elder *et al*, 2000; Lui *et al*, 2000; Uefuji *et al*, 2000; Williams *et al*, 2000; Grosch *et al*, 2001), which may result from blocking the cell cycle, enhancing c-*myc* expression, and diminishing *bcl-2* expression (Elder *et al*, 2000). In addition, among genes differentially expressed in cells treated with the specific inhibitor of COX-2, several other genes involved in the regulation of cell adhesion, cell cycle progression, apoptosis, and differentiation were found (Zhang and DuBois, 2001). While these results provided evidence that antineoplastic effects of nonsteroidal anti-inflammatory drugs (NSAIDs) may result from altered expression of genes that regulate various biological processes, the molecular mechanisms mediating antiproliferative effect of COX inhibitors are still not well defined.

Induction of cell proliferation is associated with transcriptional stimulation of growth-related genes that are required for G1/S transition (Kavin *et al*, 1997). One of them is *ornithine decarboxylase* (*odc*) gene encoding a key regulatory enzyme in the biosynthesis of polyamines that are essential for cell proliferation (Pegg, 1986). ODC and polyamines can also act as facilitating factors in triggering apoptosis (Tiberio *et al*, 2001). Thus, inhibition of ODC might be an important event among antiproliferative and proapoptotic effects of COX inhibitors.

In this study, we have examined cell proliferation, apoptosis, and ODC activity in rat hepatoma HTC-IR cells that were treated with NSAIDs, aspirin and celecoxib. In addition, an expression of immediate-early genes, c-*myc*, *Egr-1*, and c-*fos*, was assayed. We have shown that NSAIDs inhibit activity of ODC and, for the first time, differentially alter expression of c-*fos*.

## MATERIALS AND METHODS

### Cells

Rat hepatoma cells, HTC-IR, were grown in plastic cell culture flasks in DME media supplemented with 10% FBS, 2 mM glutamine, penicillin (100 U ml^−1^), streptomycin (0.01%), and humidified with 6/94% CO_2_/air gas mixture. Cells were routinely subcultured using trypsin solution. Aspirin and celecoxib were dissolved in DMSO as 1000 × stock solutions and then diluted in DMEM for the experiments. The final DMSO concentration was maintained at 0.1%.

Celecoxib was kindly provided by Pharmacia Corporation (St Louis, MO, USA).

### Cell proliferation assays

The effect of aspirin and celecoxib on HTC-IR cell growth was determined by MTT cell proliferation assay, incorporation of [^3^H] thymidine into DNA, and cell counting.

Exponentially growing cells were harvested, seeded at a density 5 × 10^3^ per well in 96-well plates and then grown in DMEM containing 10% FBS. After 24 h, fresh medium without or with aspirin or celecoxib was added. Cell growth was monitored at 24, 48, and 72 h by the cell proliferation assay, MTT CellTiter 96 (Promega). Three independent experiments were performed and all assays were repeated in octuplicate. Results were expressed as the percentage of control cells (means±s.d.).

5 × 10^3^ cells per well were seeded in 96-well plates and grown for 24 h in DMEM containing 10% FBS. Then, cells were supplemented with fresh medium containing aspirin or celecoxib or DMSO and 72 h later 0.1 *μ*Ci of [^3^H]thymidine was added to each well for an additional 4 h. Finally, cells were harvested, DNA was collected on GFC filters, and the radioactivity was determined by scintillation counting. Three independent experiments were performed and all assays were repeated in octuplicate.

Cells were seeded at a density 10^5^ cells per T25 flask. After 24 h, medium was removed, DMEM without or with aspirin or celecoxib was added and cells were incubated for 72 h. Then, floating and adherent cells were harvested and combined cell populations were counted using a haemocytometer. In addition, cell viability was evaluated by trypan blue exclusion cell staining. Results were expressed as the percentage of control cells (means±s.d.) of three independent experiments, each performed in duplicate.

### Measurement of apoptosis

Cells were grown in eight-chamber culture slides until 50% of confluency was obtained, and then were supplemented with fresh medium containing aspirin or celecoxib or DMSO. At different time points, the adherent cells were stained on chamberslides with fluorescein-conjugated annexin V and propidium iodide using Annexin-V-FLUOS Staining Kit (Roche) as per the manufacturer's instructions and the fluorescence of individual cells was assayed by fluorescence microscopy. The percentage of annexin V- and propidium iodide-stained cells within a minimum of 400 cells was determined.

### Assay of ornithine decarboxylase activity

ODC activity was assayed by determination of [^14^C]CO_2_ formation as previously described (Ostrowski *et al*, 2000). Briefly, HTC-IR cell pellets were homogenised in buffer containing 50 mM HEPES–NaOH (pH=7.5), 2.5 mM DTT, 0.25 mM pyridoxal 5-phosphate, and 0.1 mM EDTA. The reaction mixture consisted of 20 *μ*l of homogenate's supernatant, 0.25 mM pyridoxal 5-phosphate, 2.5 mM DTT, 50 mM HEPES–NaOH (pH=7.5), 0.1 mM EDTA with 0.2 *μ*Ci of L-[^14^C]ornithine hydrochloride (Amersham International) in a total volume of 40 *μ*l. The reaction tube was sealed with plastic pipette tip containing a 0.5 × 4.0 cm^2^ of Whatman No. 1 filter paper soaked with 40 *μ*l of *β*-phenylethylamine. Tubes were incubated at 37°C for 60 min and the reaction was then stopped by adding 200 *μ*l of 2 M citric acid. After further 60 min of incubation, the filter paper was removed, placed in 10 ml of scintillation liquid, and counted in a scintillation counter. Results were expressed as nmol of ^14^CO_2_ released per hour per mg of protein. All assays were performed in triplicate.

### RT–PCR

Cells were grown in T25 flasks until 60% of confluency was obtained. Then cells were made quiescent by 24 h serum deprivation, and aspirin (5 *μ*g ml^−1^) or celecoxib (5 *μ*g ml^−1^), or DMSO were added for an additional 24 h. Finally, cells were treated with 15% FBS for 0, 15, 30, 60, 180, and 360 min. Total RNA was prepared from cell pellets by acid-guanidium thiocyanate/phenol–chloroform extraction using TRIzol reagent. Five micrograms of total RNA was reverse transcribed using SuperScript II RT (GIBCO-BRL) and oligo-dT in 20 *μ*l volume as per the manufacturer's protocol. RT reactions were diluted 1 : 10 with water, and PCR was carried out using 2 *μ*l of cDNAs and primers for c-*fos*, *Egr-1*, c-*myc*, and *odc*. [*α*-^32^P]dCTP (NEN) was used to label the PCR products. PCR products were resolved on native 5% polyacrylamide gels, then the gels were dried and the PCR products were quantified using a phosphorimager. Densitometric analysis was performed using OptiQuant™ Image Analysis Software (Packard). Levels of band intensities after background subtraction were expressed in Digital Light Units (DLU).

### Statistical analysis

Results are presented as means±s.d. Significant difference between mean values was assessed by means of analysis of variance (ANOVA). *P*-values for differences from control results were calculated using Bonferroni method. Means were considered to be different if *P*<0.05.

## RESULTS

### Inhibition of cell proliferation by celecoxib and aspirin in rat hepatocytes *in vitro*

**Figure 1:**
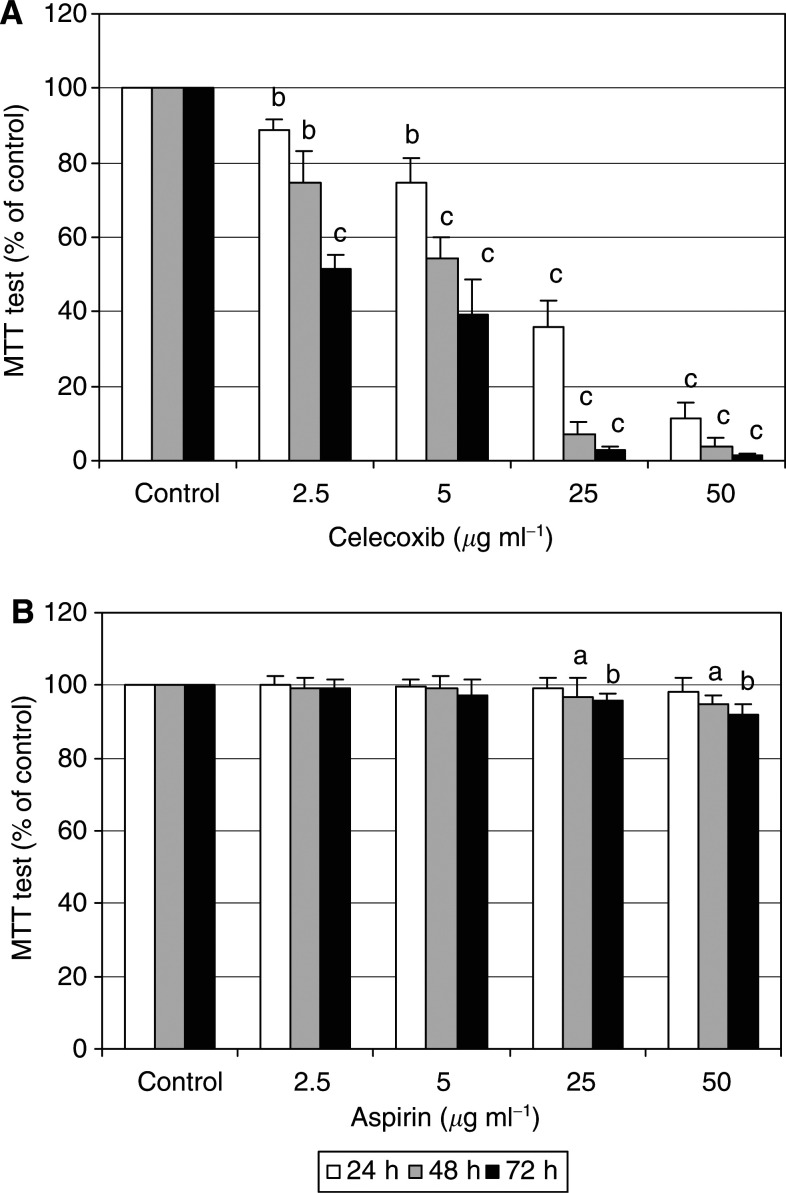
The effects of celecoxib and aspirin on proliferation of rat hepatoma HTC-IR cells determined by cell viability. Cells which were grown in 96-well plates in DMEM containing 10% FBS were treated with increasing concentrations (2.5, 5, 25, and 50 *μ*g ml^−1^) of celecoxib (**A**) or aspirin (**B**), and cell viability was monitored by MTT test 24, 48, and 72 h later. Three independent experiments were performed and all assays were repeated in octuplicate. Results are expressed as the percentage of control cell viability and represent means±s.d. a, b, c indicate significant decrease (a, *P*<0.05; b, *P*<0.01; c, *P*<0.001) in cell viability in aspirin- and celecoxib-treated cells compared to cells treated with DMSO.

MTT test reflects the combined effects of cell proliferation and survival, and the colour development results from the reduction of tetrazolium salts to formazans by living cells. HTC-IR cells were treated with increasing concentration of aspirin or celecoxib and MTT metabolisation was determined after 1, 2, and 3 days of the treatment. Celecoxib inhibited MTT metabolisation in a dose-dependent manner. The suppressive effect was observed even with the lowest COX-2 inhibitor concentration (2.5 *μ*g ml^−1^) at 24 h of the treatment, and almost complete inhibition of MTT metabolisation was seen in 48 h-treated cells with the higher concentration of celecoxib (50 *μ*g ml^−1^) (Figure 1A

). In contrast, no inhibition in MTT test was observed with low doses of aspirin (2.5 and 5 *μ*g ml^−1^), and only some inhibitory effect was observed after 48 and 72 h of the aspirin treatment with the higher drug concentrations (25 and 50 *μ*g ml^−1^) (Figure 1B).

**Figure 2:**
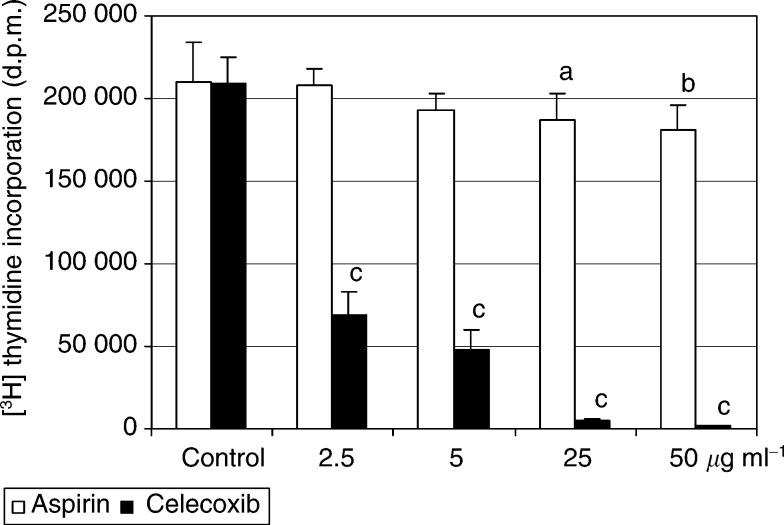
The effects of celecoxib and aspirin on proliferation of rat hepatoma HTC-IR cells determined by [^3^H]thymidine incorporation. Cells were grown in 96-well plates in DMEM containing 10% FBS. After 24 h, cells were exposed to varying concentrations (2.5, 5, 25, and 50 *μ*g ml^−1^) of celecoxib or aspirin, and 72 h later 0.1 *μ*Ci of [^3^H]thymidine was added to each well for additional 4 h. The radioactivity of collected cellular DNA was determined by scintillation counting. Three independent experiments were performed and the results represent means±s.d. of radioactivity counts expressed in d.p.m. a, b, c indicate significant decrease (a, *P*<0.05; b, *P*<0.01; c, *P*<0.001) in [^3^H]thymidine incorporation in aspirin- and celecoxib-treated cells compared to cells treated with DMSO.

**Figure 3:**
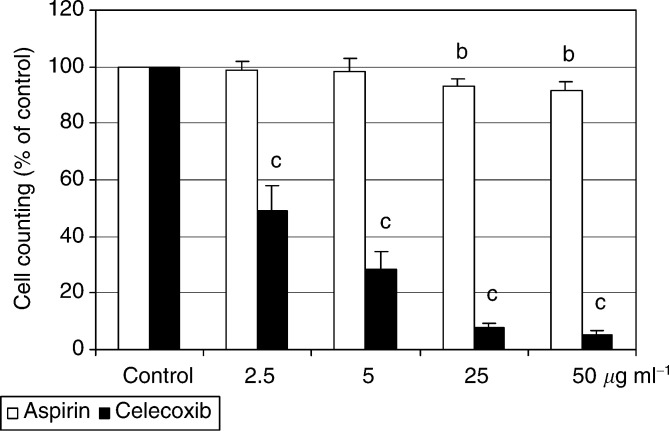
Growth inhibition of HTC-IR cells by celecoxib or aspirin treatment. Cells were grown for 72 h with or without COX inhibitors, and combined populations of floating and adherent cells were counted using a haemocytometer. Results are expressed as the percentage of control cell yield (means±s.d.) of three independent experiments, each performed in duplicate. b, c indicate significant decrease (b, *P*<0.01; c, *P*<0.001) in cell yield in aspirin- and celecoxib-treated cells compared to cells treated with DMSO.

The results of MTT test were further confirmed by two other proliferation tests. Incorporation of [^3^H]thymidine into DNA of cultured HTR-IR cells was strongly inhibited by the treatment with celecoxib and only moderately diminished by higher concentrations of aspirin (Figure 2

). Also, direct cell counting revealed that aspirin did not affect cell proliferation at low (2.5–5 *μ*g ml^−1^) concentrations (Figure 3

). However, minor but significant decline in the cell yield was evident at higher (25–50 *μ*g ml^−1^) concentrations. Over a 3-day incubated period, none of the aspirin concentration used in the study affected cell viability (as detected by trypan blue exclusion) and there was no increase in the number of nonadherent cells relative to control cells.

In contrast, celecoxib showed dose-dependent inhibitory effect on the cell yield (Figure 3). Three-day treatment with celecoxib at low (2.5–5 *μ*g ml^−1^) concentrations resulted in less than 40% of cell viability and more than 50% of floating cells; after treatment with higher celecoxib concentrations (25–50 *μ*g ml^−1^), most of the cells were floating and dead (not shown). Since the proportion of floating to adherent cells was found to be a measure of apoptosis (Eberhart and DuBois, 1995), the HTC-IR cell growth inhibition by celecoxib was likely to take place through the induction of apoptosis.

### Induction of apoptosis by celecoxib

To confirm that dominant antiproliferative effect of celecoxib is the induction of apoptosis, the quantitative analysis of apoptotic HTC-IR cells that were treated with aspirin or celecoxib was performed using fluorescence microscopy. In the early stages of apoptosis, phosphatidylserine (PS) translocates from the inner side of the plasma membrane to the external surface of the cell. Annexin V that binds to PS with a high affinity is suited to detect early apoptotic cells. Apoptotic cells in cultures undergo secondary necrosis (Vermes *et al*, 1997) and the nuclei of late apoptotic and necrotic cells can be labelled with propidium iodide. Thus, the discrimination between early apoptotic and necrotic cells was possible with the use of combined cell staining with annexin V and propidium iodide.

**Figure 4:**
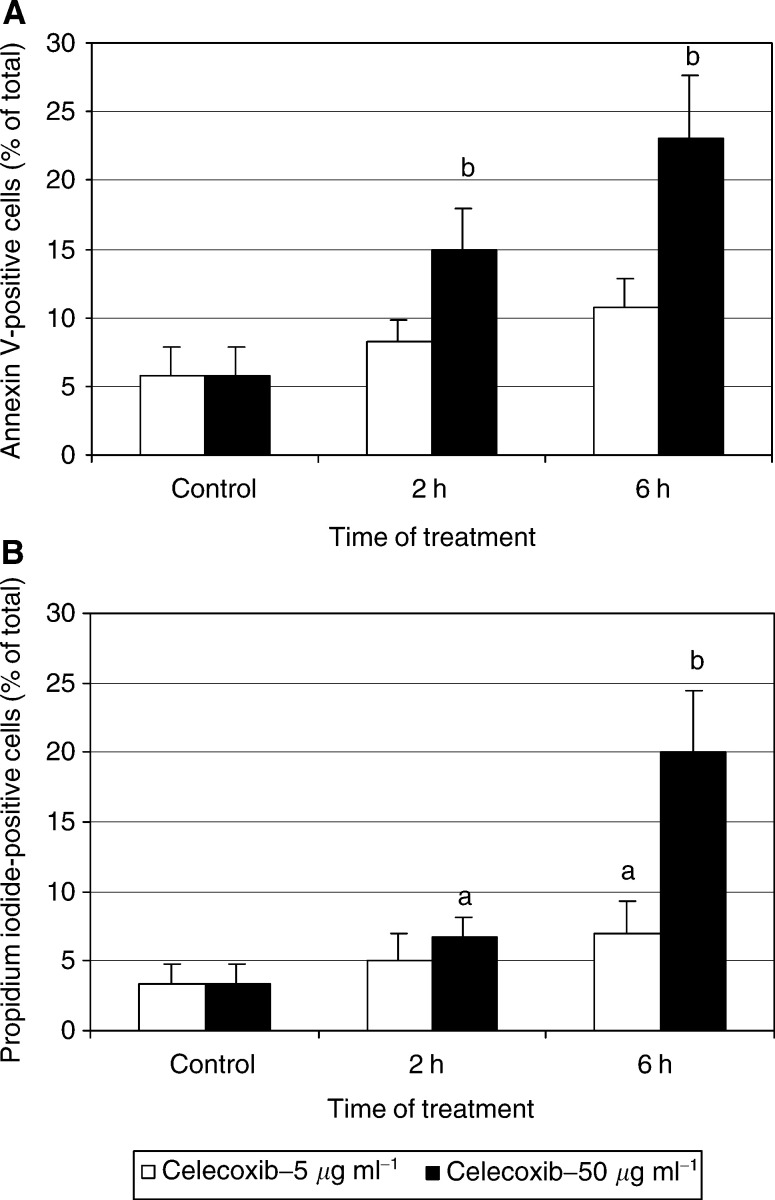
Quantification of celecoxib-induced early apoptosis (cells stained with fluorescein-conjugated annexin V) (**A**) and late apoptosis and necrosis (cells stained with propidium iodide) (**B**) in HTC-IR cells. Results represent means±s.d. of three separate experiments. a, b, c indicate significant increase (a, *P*<0.05; b, *P*<0.01; c, *P*<0.001) in number of cells stained with annexin V or propidium iodide in aspirin- and celecoxib-treated cells compared to cells treated with DMSO.

The adherent HTC-IR cells grown under standard conditions showed about 5% of total cells that were stained with annexin V, and less than 4% that were stained with propidium iodide. In repeated and quantified experiments, treatment with aspirin did not change the level of either early apoptosis or necrosis (not shown), while treatment with celecoxib caused a statistically significant, concentration- and time-dependent increase of apoptosis and necrosis (Figure 4

).

### COX inhibitors suppress ODC activity

**Figure 5:**
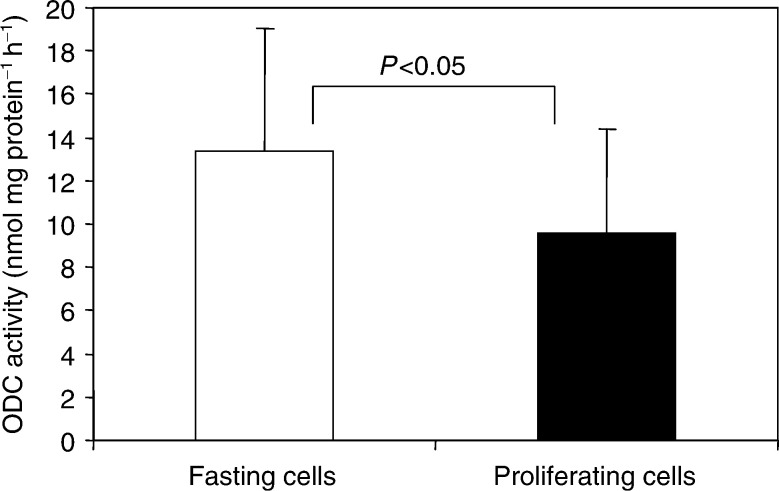
Ornithine decarboxylase (ODC) activities in growth-arrested (fasting) and proliferating HTC-IR cells. Enzyme activity was measured using L-[^14^C]ornithine hydrochloride as a substrate and expressed as nmol ^14^CO_2_ h^−1^ mg protein^−1^. Results represent means±s.d. from 14 separate experiments.

ODC activity was determined by monitoring formation of [^14^C]CO_2_ from [^14^C]ornithine. Fetal calf serum is an essential agent for cell growth in culture, supplying the cells with required proliferation signals and growth factors. Since ODC is thought to play an important role in the mitogenic responses, we determined activity of this enzyme in serum-deprived growth-arrested cells and in proliferating cells *in vitro*. As shown in Figure 5

, ODC activity was, unexpectedly, significantly higher in cells fasting for 24 h than in proliferating cells.

**Figure 6:**
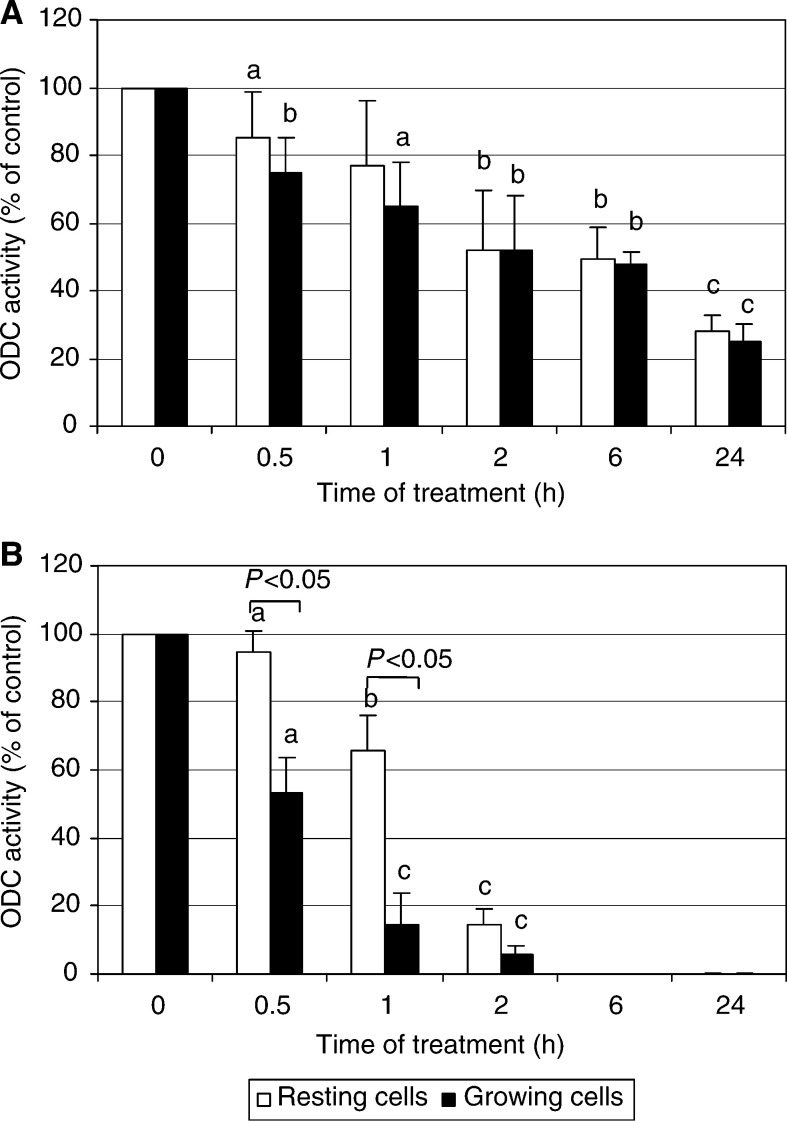
The effect of celecoxib on ODC activity. The HTC-IR cells were plated at 5 × 10^6^ per plastic cell culture flask and were grown for 24 h. Proliferating or resting (serum-starved) cells were treated with celecoxib at concentration 5 *μ*g ml^−1^ (**A**) or 50 *μ*g ml^−1^ (**B**) for 0, 0.5, 1, 2, 6, and 24 h. Then the cells were harvested and ODC activity was determined. The effects of the treatment are expressed as percentage of ODC activity in control cells treated with vehicle (0.1% DMSO) and represent means±s.d. of three separate experiments. a, b, c indicate significant decrease (a, *P*<0.05; b, *P*<0.01; c, *P*<0.001) in enzyme activity in aspirin-treated cells compared to cells treated with DMSO. *P*<0.05 indicates significant differences in ODC activity between fasting and proliferating cells.

**Figure 7:**
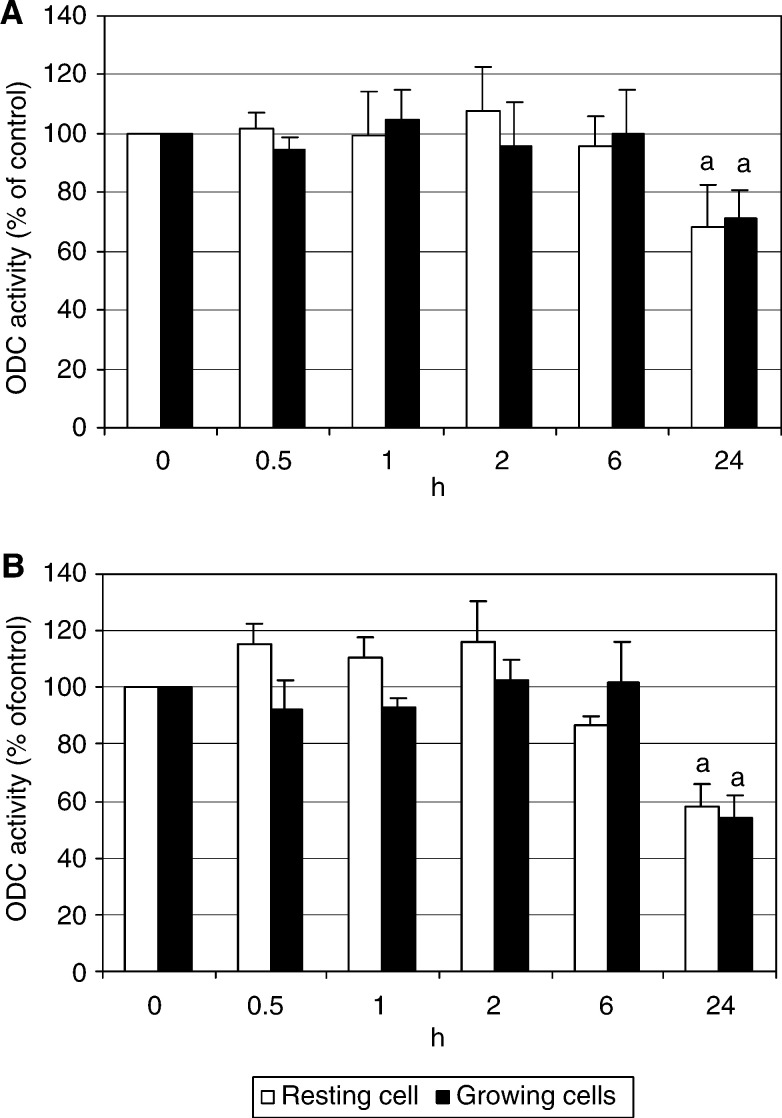
The effect of aspirin on ODC activity. Proliferating or resting HTC-IR cells were treated with aspirin at concentration 5 *μ*g ml^−1^ (**A**) or 50 *μ*g ml^−1^ (**B**). The cells were harvested at different time points and ODC activity was determined. The results are expressed as percentage of ODC activity in control cells treated with vehicle (0.1% DMSO) and represent means±s.d. of three separate experiments. a indicates significant decrease (a, *P*<0.05) in enzyme activity in aspirin-treated cells compared to cells treated with DMSO.

Treatment with celecoxib produced dose- and time-dependent decrease of ODC activity (Figure 6

). After treatment with low celecoxib concentration (5 *μ*g ml^−1^), the kinetics of enzyme inhibition was the same regardless of resting or proliferating cells were used for experiments (Figure 6A). However, at higher celecoxib concentration (50 *μ*g ml^−1^) the decrease in ODC activity was significantly higher in proliferating than in resting cells (Figure 6B). Compared to celecoxib, a much smaller degree of ODC inhibition was observed in aspirin-treated cells; until sixth hour of incubation aspirin did not inhibit ODC activity, and the significant decrease in the enzyme activity was found only at the last time point measured, 24 h (Figure 7A, B

).

### COX inhibitors do not affect expression of mRNA ODC

**Figure 8:**
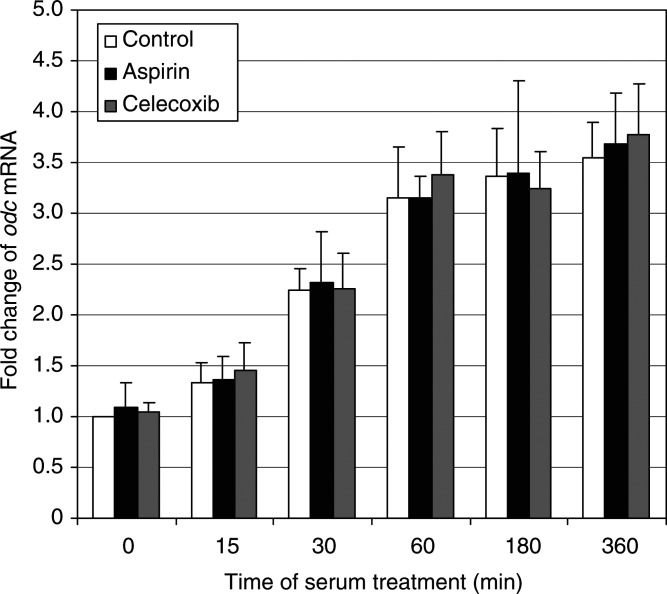
The effects of celecoxib and aspirin on the relative mRNA levels for ODC during serum stimulation. Untreated serum-starved HTC-IR cells and those treated for 24 h with 5 *μ*g ml^−1^ celecoxib or 5 *μ*g ml^−1^ aspirin were exposed to 15% FBS for 0, 15, 30, 60, 180, and 360 min. After harvesting the cells, total RNA was isolated and used in RT. PCR was carried out using [*α*^32^P]dCTP (0.5 *μ*Ci reaction^−1^) and odc primers (sense: 5′-GAGCGCTGTGACCTGCCTGA-3′; antisense: 5′-GGCAGGGTGCTGGCATCCTG-3′). PCR products were resolved by native PAGE and were quantified using a phosphorimager. Results are expressed as percentage mRNA level from serum-starved, untreated cells and represent means±s.d. of two separate experiments.

Under serum-deprived conditions, cultured cells become growth-arrested, but following serum supplementation they re-enter the cell cycle. HTC-IR cells were growth-arrested by 48-h serum starvation, and then were treated with 15% fetal calf serum. At given time points after treatment, cells were harvested, total RNA was isolated, and *odc* expression was assayed by semiquantitative RT–PCR. As shown in Figure 8

, serum treatment of starved HTC-IR cells progressively induced the expression of *odc* that achieved maximum at 6 h, the last time point measured. At this time point, *odc* was induced 3.5-fold. Pretreatment for 24 h with aspirin or celecoxib at a concentration of 5 *μ*g ml^−1^ affected neither basal nor serum-stimulated levels of mRNA ODC. Therefore, the alterations in ODC activity, described above, caused by COX inhibitors occurred through post-transcriptional events.

### COX inhibitors differentially alter gene expression

The mitogenic stimulation of various cells is accompanied by rapid induction of immediate-early genes (Herschman, 1991). We examined the effects of celecoxib and aspirin on the serum-induced gene transcription of three early genes: *Egr-1*, c-*fos*, and c-*myc*.

**Figure 9:**
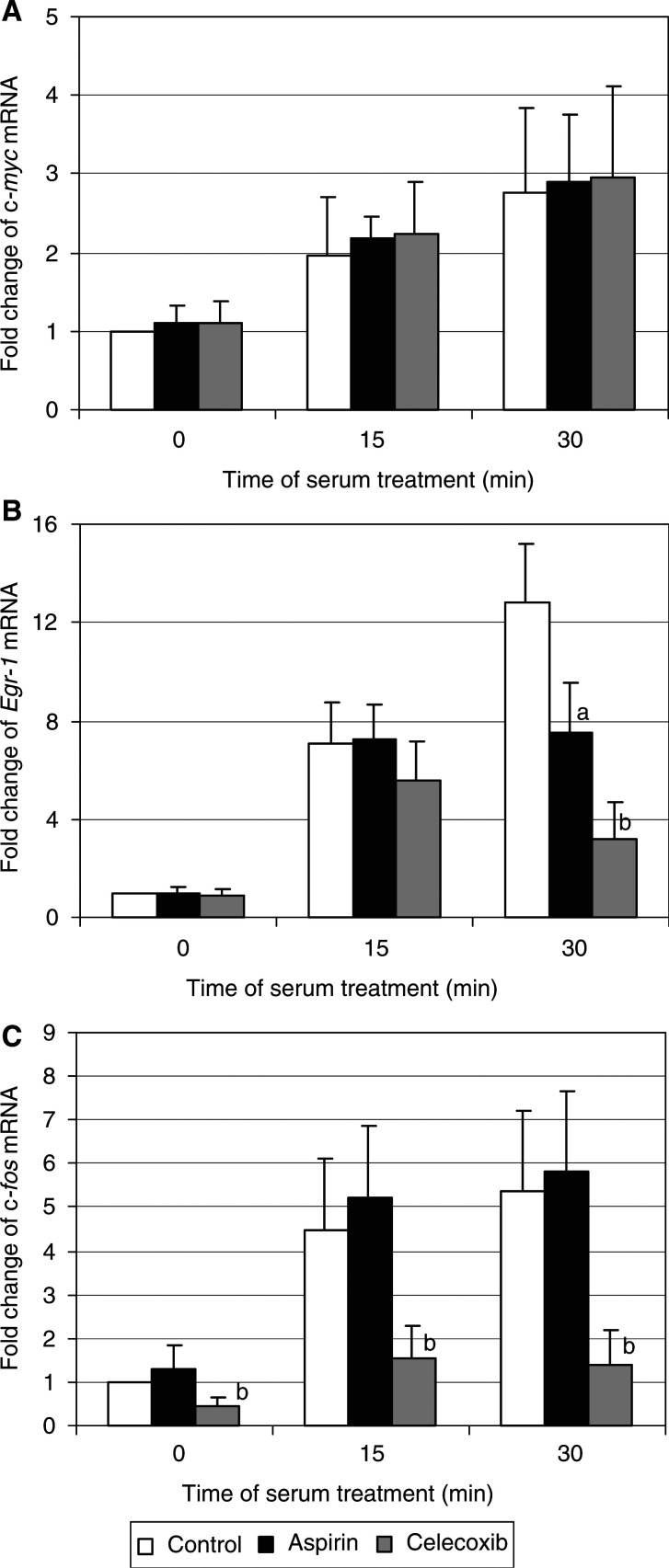
The effects of celecoxib and aspirin on the relative mRNA levels for c-*myc* (**A**), *Egr-1* (**B**), and c-*fos* (**C**) during serum stimulation. Untreated serum-starved HTC-IR cells and those treated for 24 h with 5 *μ*g ml^−1^ celecoxib or 5 *μ*g ml^−1^ aspirin were exposed to 15% FBS for 0, 15 and 30 min. After harvesting the cells, total RNA was isolated and used in RT. PCR was carried out using [*α*^32^P]dCTP (0.5 *μ*Ci reaction^−1^) and primers for c-*myc* (sense: 5′-ACGAAAAGGCCCCCAAGGTAGTT-3′; antisense: 5′-AAGGAAAAAGAAAGAAGATGGG-3′), *Egr-1* (sense: 5′-GGGGGCCCACCTACACTCC-3′; antisense: 5′-CCACCAGCGCCTTCTCGTTATTCA-3′), and c-*fos* (sense: 5′-TGCAGCTCCCACCAGTGTCTACCCC-3′; antisense: 5′-TTTGCCCCTTCTGCCGATGCTCT-3′). PCR products were resolved by native PAGE and were quantified using a phosphorimager. Results are expressed as percentage mRNA level from serum-starved, untreated cells and represent means±s.d. of six separate experiments. a, b, c indicate significant decrease (a, *P*<0.05; b, *P*<0.01; c, *P*<0.001) in mRNA levels in celecoxib- or aspirin-treated cells compared to control cells.

Semiquantitative RT–PCR analysis showed that serum treatment of starved HTC-IR cells induced the expression c-*myc*, *Egr-1*, and c-*fos* (Figure 9

). Aspirin and celecoxib did not significantly change basal and serum-stimulated c-*myc* expression (Figure 9A). Both aspirin and celecoxib changed the *Egr-1* (early growth regulated gene) expression pattern (Figure 9B). In fasting cells, and in those serum-stimulated for 15 min, the *Egr-1* mRNA levels of control and drug-treated cells were similar. However, in serum-stimulated cells for 30 min, the *Egr-1* expression was significantly lower in drug-treated cells as compared to control cells.

**Figure 10:**
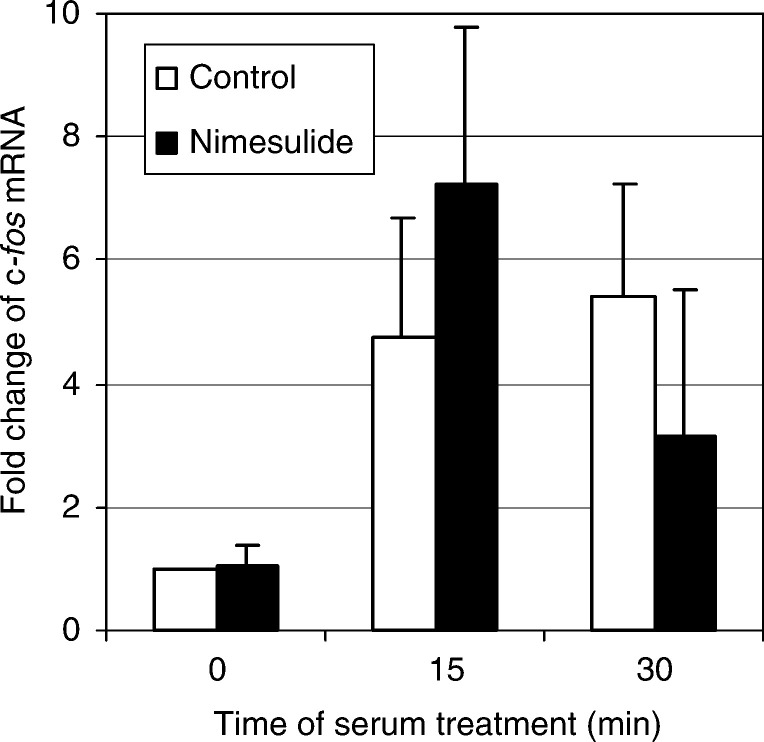
Effects of nimesulide on the relative mRNA levels for c-*fos* during serum stimulation. Untreated serum-starved HTC-IR cells and those treated for 24 h with 5 *μ*g ml^−1^ nimesulide were exposed to 15% FBS for 0, 15, and 30 min. After harvesting the cells, total RNA was isolated and used in RT–PCR. Results are expressed as percentage mRNA level from serum-starved, untreated cells and represent means±s.d. of four separate experiments.

In contrast to the gene expression pattern of *Egr-1*, expression of c-*fos* was drastically reduced by celecoxib at each time point measured, while the treatment with aspirin did not alter the levels of c-*fos* mRNA in unstimulated cells as well as in those stimulated by serum for 15 and 30 min (Figure 9C). To test whether the effect of celecoxib on c-*fos* expression might be COX-2-dependent or -independent, we examined the expression of this gene in HTC-IR cells that were treated with nimesulide, another COX-2 inhibitor. As shown in Figure 10

, nimesulide did not significantly alter both basal and serum-stimulated c-*fos* expression levels.

## DISCUSSION

This study contrasts and compares the effects of COX-1 and COX-2 inhibitors on mitogenesis, apoptosis, ODC activity, and pattern expression of selected growth-related genes in rat hepatocyte cell line, HTC-IR (expressing *COX-2* mRNA as detected by RT–PCR – not shown).

COX-2 plays the dominant role among mechanisms regulating cell survival, cell proliferation, and oncogenesis. Its induction that was observed in human carcinomas of various organs including colon, breast, prostate, lung, oesophagus, pancreas, head, neck, and brain resulted in increased cell growth, inhibition of apoptosis, activation of angiogenesis, and decreased immune responsiveness (Subbaramaiah *et al*, 1996; Zimmermann *et al*, 1999).

The final product of COX-2 is PGH_2_ that acts as the immediate precursor for other PGs and thromboxanes involved in controlling cell proliferation (Herschman, 1994). The major product of COX-2 is PGE_2_, which induces expression of Bcl_2_, inhibitor of apoptosis (Sheng *et al*, 1998). Antiproliferative effect of NSAIDs was thought to be caused predominantly by COX-2 inhibition. However, the decreased production of PGE_2_ induced by COX-2 inhibitors did not correlate with the inhibition of cell proliferation (Erickson *et al*, 1999), and exogenous PGE_2_ did not prevent the antiproliferative or proapoptotic effects of COX inhibitors (Hanif *et al*, 1996; Elder *et al*, 2000) suggesting that these processes may be unrelated. COX-2 inhibitors can reduce cell proliferation and survival also in cells that do not express COX-2 (Smith *et al*, 2000; Williams *et al*, 2000; Grosch *et al*, 2001) and cells transfected with an expression vector carrying cDNA of either COX-1 or COX-2 possessed a similar stimulation of growth rates (Williams *et al*, 2000). Therefore, both COX-2-dependent and -independent mechanisms may contribute to antiproliferative and proapoptotic effects of NSAIDs.

Celecoxib is a specific inhibitor of COX-2, inhibiting this enzyme at concentration of 10 *μ*M. In contrast, aspirin acts as nonspecific COX inhibitor; at 10 *μ*M it inhibits COX-1 while at a concentration higher than 100 *μ*M inhibits also COX-2 (Dermott *et al*, 1999). In our experiments, aspirin at the inhibitory concentrations for COX-1 (2.5 and 5 *μ*g ml^−1^) did not exhibit any effect on HTR-IR cell proliferation. In contrast, celecoxib even at low concentrations (2.5 and 5 *μ*g ml^−1^) drastically inhibited proliferation of these cells. The extent of growth inhibition by celecoxib is related, at least in part, by proapoptotic effect induced by COX-2 inhibitors. As reported recently by others (Kusunoki *et al*, 2002; Yamazaki *et al*, 2002), the proapoptotic effect of celecoxib seems to be unrelated to the inhibition of COX-2.

ODC is the first and rate-limiting enzyme in the polyamine pathway and, therefore, it is a key regulatory enzyme in growth processes (Pegg, 1986). The enzyme function is to convert ornithine to putrescine, and changes in ODC activity reflect the rate of macromolecular synthesis. Cancer cells display upregulation of ODC, and sustained high level of ODC activity is implicated as an essential component of tumour development. Indeed, increased expression of ODC mRNA and high activity of ODC were observed in rat (Huber *et al*, 1989; Ostrowski *et al*, 2000) and human hepatocellular carcinoma (Tamori *et al*, 1994). ODC activity correlated also with growth rate in a panel of hepatoma cell lines (Williams-Ashman *et al*, 1972). However, we have found that ODC activity in growth-arrested HTC-IR cells was significantly higher than in exponentially growing cells. These results, although unexpected, could indicate that under 24-h serum-deprived conditions, most of HTC-IR cells were rather in G_1_ than in G_0_ phase of the cell cycle. It is known that ODC induction and putrescine accumulation occur in G_1_ and G_2_ phases of the cell cycle, whereas spermine and spermidine accumulate in S phase along with RNA and DNA synthesis (Heby and Persson, 1990).

Treatment with celecoxib produced dramatic decrease in ODC activity. Much lesser inhibitory effect on ODC activity was observed in aspirin-treated cells. Since COX inhibitors did not diminish the transcription of *odc*, the inhibition of ODC activity resulted from post-transcriptional enzyme modification. At high concentration, celecoxib decreased activity of ODC in proliferating cells stronger than in growth-arrested cells suggesting that this inhibitory effect of celecoxib may depend on the phase of cell cycle. In contrast, the inhibitory effect of aspirin did not correspond to proliferative state of the cell. In regard to these findings, inhibition of ODC activity by COX inhibitors may be a consequence of at least dual events: COX-2 inhibitory-dependent cell death and some unknown COX-independent mechanism(s). In fact, as reported recently (Turchanowa *et al*, 2001), treatment of colon cancer cells with indomethacin (a nonselective COX-1 and COX-2 inhibitor) induced an irreversible cascade of events leading to oxidative stress, activation of spermidine/spermine-acetyltransferase (SSAT), polyamine depletion, and cell death. Thus, decreased ODC protein level and its enzymatic activity caused by indomethacin were rather a consequence of impaired proliferation than a direct effect of indomethacin on ODC protein.

Growth signals are linked to gene expression. Immediate-early genes are activated in a protein synthesis-independent manner and are involved in cell proliferation, differentiation, apoptosis, and oncogenic transformation (Jochum *et al*, 2001). They regulate later phases in G_1_ of the cell cycle and represent diverse functional classes including transcription factors. In this study, we determined the expression of three immediate-early genes encoding transcription factors, c-*myc*, *Egr-1*, and c-*fos*. c-*myc* target genes include several genes involved in growth control and therefore c-*myc* is implicated as a direct regulator of cell cycle machinery (Schmidt, 1999). One of c-*myc*-dependent genes is *odc* (Bello-Fernandez *et al*, 1993). During normal mitogenesis c-*myc* expression transiently increases in G_1_. *Egr-1* is a zinc-finger transcription factor that is rapidly activated by a variety of extracellular signals or tissue injury (Szabo *et al*, 2001). Members of Fos family of transcription factors are thought to have a primary function in activating transcription of delayed-early genes expressed subsequently in the growth response (Taub, 1996).

c-*fos*, *Egr-1*, and several other immediate-early genes contain in their promoter region serum response element (SRE) that is rapidly activated in response to serum treatment. Serum activates SRE-driven promoters via Ras-MAP kinase pathway (Tice *et al*, 2002). In our study, as expected, following serum readmission to the serum-deprived HTC-IR cells, the expression of the immediate-early genes significantly increased at 15–30 min.

Treatment of cells with aspirin or celecoxib did not alter basal and serum-stimulated c-*myc* expression. Also, the treatment of rats with the selective COX-2 inhibitor, NS-398 did not change c-*myc* mRNA levels in colon mucosa as compared to untreated animals, although in rat treated with a colon-specific carcinogen, azoxymethane, NS-398 significantly decreased the expression of c-*myc* elevated by carcinogen (Kishimoto *et al*, 2002). Thus, neither the short-term treatment *in vitro* nor the long-term treatment *in vivo* with COX-2 inhibitors altered c-*myc* expression. In contrast, indomethacin can reduce c-*myc* protein level in colon cancer cells (Turchanowa *et al*, 2001). Since COX inhibitors did not alter the transcription of *odc*, it is unlikely that the inhibition of ODC activity might be a consequence of a direct relation between c-*myc* and ODC.

Both aspirin and celecoxib inhibited serum-induced *Egr-1* mRNA levels and these results are consistent with the inhibitory effect of indomethacin and NS-398 on vascular endothelial growth factor-stimulated expression of *Egr-1* in human microvascular endothelial cells (Szabo *et al*, 2001).

While c-*fos* expression was drastically reduced by celecoxib at each time point measured, aspirin treatment did not alter the levels of c-*fos* mRNA in fasting and serum-stimulated cells. This inhibitory effect of celecoxib on c-*fos* expression seems to be a celecoxib-specific and COX-2-independent since the treatment with nimesulide, another highly selective COX-2 inhibitor, did not significantly alter both basal and serum-stimulated c-*fos* expression levels.

A distinct subset of genes is regulated by the COX-2 inhibitor (Zhang and DuBois, 2001), and alteration in gene expression programming could be a target in the anticancer activity of COX inhibitors. The diminishing of *Egr-1* and c-*fos* by celecoxib may reduce their target gene activation involved in controlling cellular proliferation. However, changes in the array of gene expression might not be a cause of antiproliferative and/or aproapoptotic effects of NSAIDs if other genes could compensate the downregulation of the immediate-early genes.

In summary, we have shown that the treatment of HTC-IR hepatoma cells with the selective COX-2 inhibitor, celecoxib, was associated with induction of apoptosis and complete inhibition of cell proliferation, suppression of ODC activity, and diminished c-*fos* expression. Aspirin exhibited a small antiproliferative effect with moderate inhibition of ODC activity that was not associated with the proapoptotic effect or alteration in c-*fos* expression. Both inhibitors significantly decreased the expression of *Egr-1*. Although celecoxib and aspirin share the ability to inhibit one or both COX isoforms, the molecular mechanisms of antiproliferative and proapoptotic effects of NSAIDs are still not well understood. Some of the observed effects are likely to be related to COX-independent pathways and may be drug-specific. Thus, the precise mechanisms of NSAIDs action should be defined before using these drugs for cancer chemopreventive therapy.
